# Substrate Type Influences the Structure of Epiphyte Communities and the Growth of *Posidonia oceanica* Seedlings

**DOI:** 10.3389/fpls.2021.660658

**Published:** 2021-05-07

**Authors:** Davide De Battisti, Elena Balestri, Giuseppina Pardi, Virginia Menicagli, Claudio Lardicci

**Affiliations:** ^1^Department of Biology, University of Pisa, Pisa, Italy; ^2^Department of Earth Science, University of Pisa, Pisa, Italy; ^3^Centre for Climate Change Impact, University of Pisa, Pisa, Italy

**Keywords:** seagrass, recruitment, seedling, substrate, epiphyte, biological interaction

## Abstract

Epiphytes colonizing adult seagrasses highly contribute to seagrass ecosystem functioning and plant growth. Yet, little information exists on epiphytic communities developing on seagrass seedlings. Moreover, for some species our knowledge about seedling performance is limited to early establishment phases, and the role of substrate type in affecting their growth is still unclear. These are considerable knowledge gaps, as seedlings play an important role in meadow expansion and recovery from disturbance. In this study, seedlings of *Posidonia oceanica*, a keystone species of the Mediterranean, were grown in a shallow (1.5 m deep) coastal area along the Tuscany coast (Italy). After five years of growth (July 2009), seedlings were collected and, through multivariate analysis, we examined whether the epiphytic communities of leaves (both internal and external side) and rhizomes, as well as the growth characteristics differed between rock and sand substrate. The epiphytic communities of seedlings largely reflected those found on adult shoots. Epiphyte cover was similar between the two leaf sides, and it was higher on seedlings grown on rock than on sand, with encrusting algae dominating the community. No differences in epiphyte cover and community structure on rhizomes were found between substrates. Seedling growth characteristics did not differ between substrates, apart from the number of standing leaves being higher on rock than on sand. No correlation was found among epiphyte communities and seedling growth variables (i.e., leaf area, maximum leaf length, number of leaves, total number of leaves produced, rhizome length, total biomass, and root to shoot biomass ratio). Results indicate that epiphytes successfully colonize *P. oceanica* seedlings, and the surrounding micro-environment (i.e., substrate type) can influence the leaf epiphytic community. This study provides new valuable insights on the biological interactions occurring in seagrass ecosystems and highlights the need for better understanding the effects of seedling epiphytes and substrate on the formation of new meadows.

## Introduction

Seagrasses host a variety of epiphytic organisms, from cyanobacteria to macroalgae and invertebrates (Piazzi et al., 2007; Uku et al., 2007; Wilson, 2007), which strongly contribute to meadow productivity and biodiversity, and sustain sediment formation and food web (Boudouresque, 2004; Borowitzka et al., 2006; Michael et al., 2008; Piazzi et al., 2016). Epiphytes can also influence seagrass growth both negatively, competing for light and nutrients (Alcoverro et al., 2004; Borowitzka et al., 2006; Nelson, 2016), and positively, protecting leaves from photo-inhibition and desiccation, and providing nitrogen to plants (Harlin, 1975; Orth and Van Montfrans, 1984). Therefore, identifying the factors structuring epiphyte communities is fundamental for improving our understanding of seagrass ecosystems and establishing more efficient management strategies.

The epiphyte community that develops on adult seagrass plants arises from the combination of multiple biological and environmental factors, including plant growth cycle, light and nutrient availability, hydrodynamic regimes, temperature, and the nature of the substrate (Borowitzka et al., 2006; Michael et al., 2008; Ben Brahim et al., 2014b, 2020; Mabrouk et al., 2014). Since many algal epiphytes preferentially grow under high light availability conditions (Borowitzka et al., 2006), they are generally more abundant in plants growing in shallow than in deep meadows (Pinckney and Micheli, 1998; Rindi et al., 1999) and on the external leaf side of older leaves (Trautman and Borowitzka, 1999; Piazzi et al., 2016). Furthermore, species such as *Posidonia oceanica* L. Delile harbor higher epiphytic abundance and diversity in summer than in winter (Piazzi et al., 2016). Also, substrate type can strongly influence the epiphyte recruitment on seagrasses because rocky substrates provide a larger number of propagules (Van Elven et al., 2004; Borowitzka et al., 2006). Indeed, *Thalassodendron ciliatum* (Forsk.) den Hartog and *P. oceanica* harbor a higher epiphyte abundance on plants growing on hard substrates in comparison to soft bottoms (Bandeira, 2002; Piazzi et al., 2016), although for the latter this applies to rhizomes (Piazzi et al., 2016) but not leaves (Giovannetti et al., 2008).

Surprisingly, our knowledge of seagrass epiphytes is based on adult plants, but very little is known about the structure of epiphyte communities on seedlings. Available data are currently limited to 6-month old *P. oceanica* seedlings and mainly concerned the effects of a species-specific bacterium on epiphyte recruitment (Celdran et al., 2012). Thus, it is largely unknown whether other factors structuring the epiphyte communities of adult plants (e.g., substrate type) are also acting at the seedling stage. This is a relevant gap in knowledge considering the critical role that sexual recruitment plays in the maintenance and expansion of seagrass meadows (Balestri and Lardicci, 2008; Kendrick et al., 2012; Tan et al., 2020; Vanderklift et al., 2020).

*Posidonia oceanica* is an endemic, keystone seagrass of the Mediterranean Sea that forms extensive meadows on a variety of substrates (e.g., sand and rock) up to 45 m depth (Procaccini et al., 2003). This species provides important ecosystem services (e.g., coastal protection and nursery for fish; Campagne et al., 2015), and support a highly diverse epiphytic community (more than 600 species; Mazzella et al., 1989, 1992; Piazzi et al., 2016). Unfortunately, *P. oceanica* meadows are declining in the entire Mediterranean basin (Telesca et al., 2015) and, therefore, there is a strong interest in improving the conservation status of existing meadows.

Recent studies have shown that recruitment by seeds play an important role in the colonization of new sites, maintenance of genetic diversity, and for meadows recovery after disturbance (Balestri and Vallerini, 2003; Balestri and Lardicci, 2008; Alagna et al., 2013; Balestri et al., 2017). Furthermore, seedlings are a promising plant material for restoration interventions (Balestri et al., 1998; Terrados et al., 2013). Naturally established seedlings have been found in sheltered, shallower areas (<3 m depth) on different substrate types, preferentially on rock and sand substrate (Balestri et al., 2017; Pereda-Briones et al., 2020). Substrate type could influence seedlings development, as suggested by experimental studies where seedlings preferentially invested on shoots or roots depending if they grew on hard or unconsolidated substrate (i.e., rock and sand; Alagna et al., 2013, 2015). However, all available data on seedling performance are restricted to their first three years of life (Balestri et al., 1998; Balestri and Bertini, 2003; Alagna et al., 2013) and no data are available about their epiphytic community at this stage.

Here, we investigated the epiphytic community of leaves and rhizomes of *P. oceanica* seedlings grown for five years in a shallow site on two substrate types (rock *vs.* sand). We also examined seedling growth rate and biomass allocation. We hypothesized that seedlings grown on rock and sand differed in both total epiphyte cover and community structure, as well as in growth pattern. Furthermore, previous studies conducted on *P. oceanica* adult shoots have shown differences in epiphyte cover between the internal and the external leaf sides (Montefalcone et al., 2006), therefore, we hypothesized that the same epiphyte distribution pattern may occur on leaves of seedlings.

## Materials and Methods

### Experimental Procedure

**FIGURE 1:**
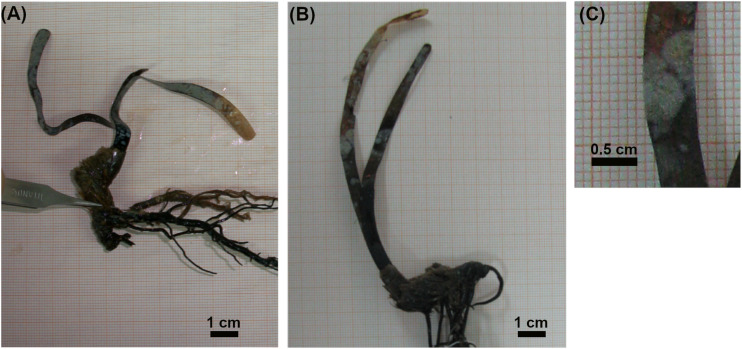
Images of five-year old *Posidonia oceanica* seedlings grown on rock **(A)** or sand **(B)** substrate, and of epiphytes grown on leaves **(C)**.

Seedlings of *P. oceanica* were harvested five years after their transplanting at 1.5 m depth in a sheltered site in the Ligurian Sea (Livorno, Italy, 43 29 N, 1019 E; Supplementary Figure 1), near to a *Cymodocea nodosa* bed; no adult plants of *P. oceanica* were present close to transplanted seedlings (the closest *P. oceanica* meadow was >5 m deep). The substrate consisted of rock and patches of medium-fine calcareous sand (Balestri et al., 2015). The transplantation procedure has been described in a previous study (Balestri et al., 2015). Briefly, *P. oceanica* fruits were collected on a beach near Livorno (Italy) in June 2004, and seeds were extracted from fruits and placed in plastic boxes floating in an aquaculture tank equipped for seagrass culture (Balestri and Lardicci, 2012; Balestri et al., 2017). After one month, seedlings were transferred to the transplanting site where they were individually planted in rock fissures and sand patches; rock and sand patches were approximately 15 m apart, while the distance between seedlings was at least 50 cm. Rocks were colonized by turf species mainly consisted in filamentous algae, while sand patches were bare (Balestri et al., 2015). In July 2009, five seedlings (Figure 1) grown on rock and five seedlings grown on sand were carefully collected by hand and transported to the laboratory for plant phenology and epiphyte community analyses.

### Seedling Growth Variables and Epiphyte Characterization

Collected seedlings were washed from sediment and separated into aboveground structures (leaves, leaf sheaths, and rhizome) and belowground structure (roots). The two oldest leaves of each seedling were used for leaf epiphyte community characterization. Both the internal and external sides of each leaf were visually examined, in entirety, under a stereomicroscope equipped with a micrometer (Balata et al., 2007; Giovannetti et al., 2010; Mabrouk et al., 2017). Organisms (invertebrates and algae) were identified at species level when possible, otherwise higher taxonomical categories were used. To evaluate the abundance of each organism, the surface area (cm^2^) covered by each organism in orthogonal projection on the leaf was recorded (Pardi et al., 2006). Afterward, for each organism we calculated the percentage area covered with respect to total leaf area (Balata et al., 2010; Mabrouk et al., 2017). Similarly, the epiphyte community present on the rhizome of each seedling was examined. Total epiphyte percentage cover on rhizome was calculated by summing the percentage cover of each organism found on each of the two sides of the rhizome. Furthermore, for both leaves and rhizomes algae were grouped into morphological groups according to the classification of Steneck and Dethier (1994).

Then, for each seedling we counted the number of standing leaves, the length of the longest leaf, and the number of rhizome internodes. The total number of leaves produced by each seedling during its life span was obtained by summing the number of internodes and the number of standing leaves. For each seedling, the length and the width of standing leaves were measured using a ruler; leaf area was calculated multiplying leaf length by width and summing the areas of all leaves (Drew and Jupp, 1976). Rhizome length of each seedling was measured using a binocular stereomicroscope equipped with a micrometer. All separated seedling portions were oven-dried at 70C to constant weight and weighted. Total plant biomass was determined by summing the biomass of above- and belowground portions, and the root to shoot biomass ratio was also calculated.

### Statistical Analysis

A mixed-effect model (lme4 package; Bates et al., 2015) was used to test for differences in total leaf epiphyte cover between substrate types and leaf surface sides. In this model, total epiphyte cover was included as the response variable and substrate type (two levels: rock and sand) and leaf side (two levels: internal or external) as the predictor variables; shoot was considered as a random factor nested within substrate type to account for the non-independence of the two sides of the individual leaf. A two-sided *t*-test was used to investigate whether total epiphyte percentage cover on rhizomes differed between substrate types, i.e., rock and sand.

We used the multivariate analysis of variance based on permutations (PERMANOVA; Anderson, 2001) to test for differences between substrates in the epiphyte percentage cover on rhizomes and leaves, as well as in the percentage cover of algal morphological groups on leaves. Data from both sides of leaves were averaged since no differences were found in total epiphyte cover between the two leaves. PERMANOVAs was performed using the adonis function in the vegan package (Oksanen et al., 2019), setting 999 permutations for the test at an -level of 0.05.

To visualize the epiphyte community composition on leaves and rhizomes of *P. oceanica* grown on the two substrates we used a two-dimensional n-MDS ordination (non-metric multidimensional scaling; Clarke, 1993). SIMPER analysis was used for identifying which organism or morphological group primarily accounted for observed differences in epiphytic assemblages between substrate types.

Separate two-sided *t*-tests were used to test for differences in growth characteristics of seedlings grown on the two substrate types. We used two-sided *t*-tests because of the scarcity of information on epiphytes growing on seedlings. Lastly, the RELATE procedure (PRIMER v.7) was used to assess possible correlations between seedling growth variables and leaf epiphyte cover. Separate analyses were performed for seedlings grown on sand and rock substrate. In this procedure, the matrix of similarities between epiphytic species abundance (based on the Bray Curtis coefficient from four root transformed data) was compared with a matrix of the similarities between morphological seedling variables (based on Euclidean distance from normalized data), and the significance of any correlation between the matrices was computed with a randomization test.

Before performing the analyses, ShapiroWilk and Levenes test were used to test for assumptions of normality and homogeneity of variance. In the mixed-effect model, the response variable was square root transformed to meet model assumption. In PERMANOVA, a BrayCurtis dissimilarity matrix (Bray and Curtis, 1957) calculated from fourth root transformed data was used to meet the assumption of homogeneity of variance. The permutest function (vegan package) was used to check for between groups dispersion. For morphological groups in rhizomes, a constant of 1 was added to avoid NAs generation. All plots were made using the ggplot2 package (Wickham, 2016) and all analysis were performed in R (R Core Team, 2018) except for the RELATE procedure that was performed in PRIMER (Anderson et al., 2008; Clarke and Gorley, 2015).

## Results

In total, 13 species of macroalgae (eight Rhodophyta, three Heterokontophyta, and two Chlorophyta) and five taxa of invertebrates (two Hydroidea, one Polychaeta, one Bryozoan, and one Foraminifera; Table 1) were identified on leaves and rhizomes of *P. oceanica* seedlings. Seedlings from the two substrates shared only few taxa or species: encrusting algae, *Chondria dasyphylla*, *Sphacelaria cirrosa*, foraminifera, and *Spirorbis* spp. (Table 1). Encrusting algae comprised species belonging to the Corallinaceae family such as *Pneophyllum fragile*, *Hydrolithon farinosum*, *Hydrolithon cruciatum*, and *Hydrolithon boreale*. Exclusives of seedlings from rock were *Chondria mairei*, *Dictyota fasciola*, *Herposiphonia tenella*, *Vertebrata subulifera*, and *Obelia caniculata*, whereas *Ceramium* sp., *Herposiphonia secunda*, *Polysiphonia* sp., *Dictyota mediterranea*, and *Orthopyxis caniculata* were present only on seedlings from sand (Table 1).

**TABLE 1:** Species and taxa found on leaves and rhizomes of five-year old *Posidonia oceanica* seedlings growing on rock or sand substrate.

	**Rock**	**Sand**	**Morphological group**
*Leaf*			
**Rhodophyta**			
*Ceramium spp.*	No	Yes	Filamentous
*Chondria dasyphylla* (Woodward) (C. Agardh)	Yes	Yes	Corticated
*Chondria mairei* Feldmann-Mazoyer	Yes	No	Corticated
*Herposiphonia secunda* (C. Agardh) Ambronn f. *secunda*	No	Yes	Filamentous
*Herposiphonia secunda* (C. Agardh) Ambronn f. *tenella* (C. Agardh) M.J. Wynne	Yes	No	Filamentous
*Hydrolithon farinosum* (J. V. Lamouroux) Penrose et Y. Chamberlain	Yes	Yes	Encrusting
*Pneophyllum fragile* Kutzing	Yes	Yes	Encrusting
*Polysiphonia spp.*	No	Yes	Filamentous
*Vertebrata subulifera* (C. Agardh) Harvey	Yes	No	Filamentous
**Heterokontophyta**			
*Dictyota fasciola* (Roth) J.V. Lamouroux	Yes	No	Foliose
*Dictyota mediterranea* (Schiffner) G. Furnari	No	Yes	Foliose
*Sphacelaria cirrosa* (Roth) C. Agardh	Yes	Yes	Filamentous
**Hydroidea**			
*Obelia geniculata* (Linnaeus, 1758)	Yes	No	
*Orthopyxis caliculata* (Hincks, 1853)	No	Yes	
**Foraminifera**	Yes	Yes	
**Polychaeta**			
*Spirorbis spp.* Daudin, 1800	Yes	Yes	
*Rhizome*			
**Rhodophyta**			
*Chondria dasyphylla* (Woodward) (C. Agardh)	Yes	Yes	Corticated
*Herposiphonia secunda* (C. Agardh) Ambronn f. *secunda*	Yes	Yes	Filamentous
*Herposiphonia secunda* (C. Agardh) Ambronn f. *tenella* (C. Agardh) M.J. Wynne	Yes	No	Filamentous
*Hydrolithon farinosum* (J. V. Lamouroux) Penrose et Y. Chamberlain	Yes	No	Encrusting
*Jania virgata* (Zanardini) Montagne	Yes	No	Articulate
*Lophosiphonia obscura* (C.Agardh) Falkenberg	Yes	No	Filamentous
*Pneophyllum fragile* Kutzing	Yes	No	Encrusting
*Polysiphonia spp.*	No	Yes	Filamentous
**Heterokontophyta**	No	No	
*Dictyota mediterranea* (Schiffner) C. Agardh	No	Yes	Foliose
*Sphacelaria cirrosa* (Roth) C. Agardh	Yes	Yes	Filamentous
**Chlorophyta**			
*Anadyomene stellata* (Wulfen) C. Agardh	Yes	No	Foliose
*Valonia utricularis* (Roth) C. Agardh	Yes	No	Encrusting
**Foraminifera**	No	Yes	
**Polychaeta**	Yes	No	
*Spirorbis spp.* Daudin, 1800	Yes	Yes	
**Bryozoa**			
*Amanthia lentigera* (Linnaeus, 1761)	Yes	No	

**FIGURE 2:**
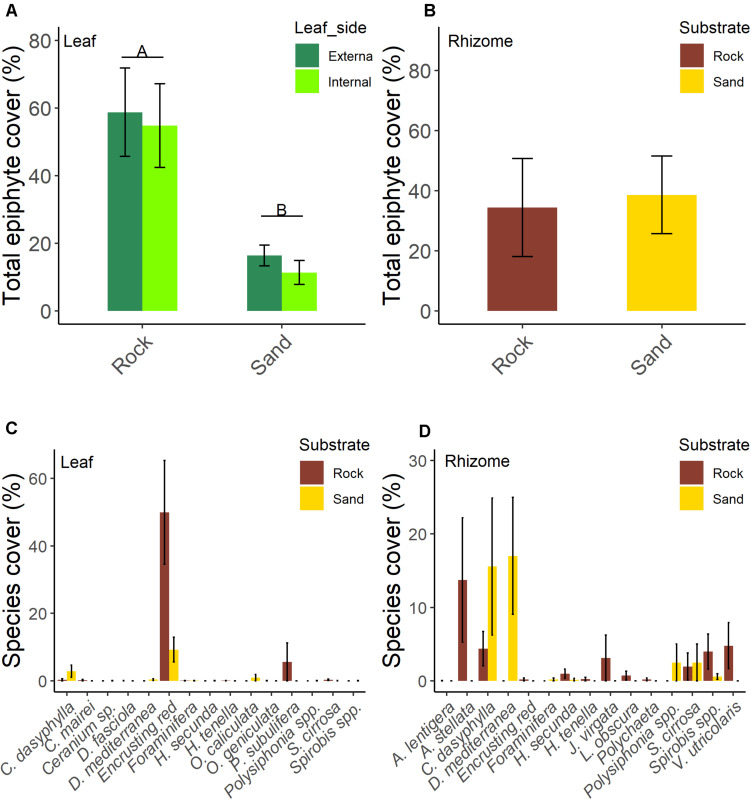
Total epiphytic percentage cover of the two sides of leaves **(A)** and rhizomes **(B)** and epiphytic percentage cover of organisms of leaves [data are averaged for leaf sides; **(C)**] and rhizomes **(D)** of five-year old seedlings of *Posidonia oceanica* grown on rock and sand substrate. Letters above bars denote significant differences between treatments at < 0.01. Data are means SE; *n* = 5.

No significant differences in the total leaf epiphyte cover between the internal and external side of leaves were found (Table 2). Substrate type significantly affected leaf epiphyte cover (Table 2 and Figure 2A); mean percentage cover was higher on rock (57.2% 8.5, mean SE) than on sand (13.4% 2.6 SE). Encrusting algae dominated the epiphyte assemblages on leaves in both substrates type, followed by *V. subulifera* and *C. dasyphylla* (Figure 2C).

**TABLE 2:** Mixed-effect model results of the effect of substrate type (two levels, rock and sand) and leaf side (two levels, internal and external) on total leaf epiphyte cover (expressed in percentage) on five-year old seedlings of *Posidonia oceanica*.

**Source**	**Estimate**	**Standard error**	**df**	***t*-value**	***p*-value**	**mR^2^**	**cR^2^**
(Intercept)	7.502	0.679	10.15	11.052	**<0.001**	0.63	0.91
Substrate: sand	3.592	0.960	10.15	3.741	**<0.01**		
Leaf side: Internal	0.258	0.471	8	0.548	0.598		
Substrate x Leaf side: Internal	0.494	0.666	8	0.741	0.479		

*In bold significant effect. Marginal R^2^ = mR^2^; conditional R^2^ = cR^2^.*

For rhizomes, the total cover of epiphytes was similar (*t* = 0.201, df = 8, *p* = 0.846; Figure 2B) between rock (34.4% 16.4 SE) and sand (38.6% 12.9 SE). The most represented species or taxa on rhizomes (Figure 2D) were *Anadyomene stellata*, *C. dasyphylla*, *D. mediterranea*, *Spirobis* spp., *Polysiphonia* spp., *S. cirrosa*, and *Valonia utricolaris*.

**FIGURE 3:**
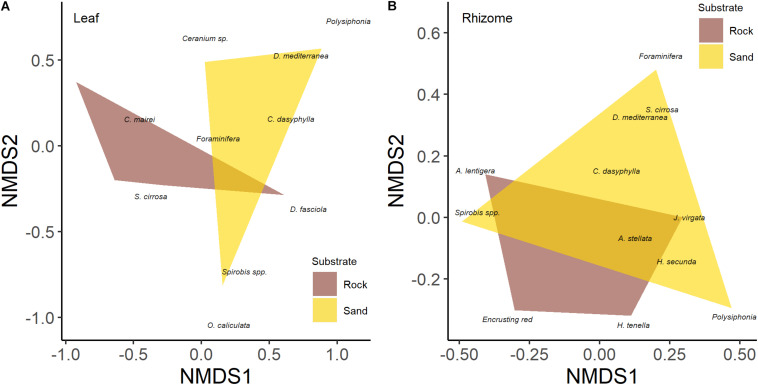
Differences in epiphyte community of leaves **(A)** and rhizomes **(B)** of five-year-old seedlings of *Posidonia oceanica* grown on rock and sand substrate type based on n-MDS classification.

Multivariate analysis detected a significant effect of substrate type on the leaf epiphyte community (data were averaged for leaf sides; Table 3) which was also shown by the n-MDS configuration of the two communities (Figure 3A). SIMPER analysis showed that encrusting algae contributed the most to differences in communities of leaves between substrates (74%), followed by *V. subulifera* (14%), and *C. dasyphylla* (6%; Table 4). However, no significant difference in total epiphyte percentage cover on rhizomes between the two substrates was found (Table 3). Indeed, n-MDS configuration shows an overlap between the epiphyte communities of rhizomes from the two substrate types (Figure 3B).

**TABLE 3:** Results of multivariate analysis of the epiphyte community (at the level of taxa and algal morphological groups) on leaves and rhizomes of five-year old *Posidonia oceanica* seedlings grown on rock and sand substrates.

	**Source**	**d.f.**	**Sum of squares**	**Mean squares**	**Pseudo-F**	**p-value**	**R^2^**
**Taxa or species**							
*Leaf*							
	Substrate type	1	0.751	0.751	4.519	**0.013**	0.36
	Residuals	8	1.330	0.166			
	Total	9	2.081				
*Rhizome*							
	Substrate type	1	0.6401	0.641	1.677	0.94	0.17
	Residuals	8	3.057	0.382			
	Total	9	3.697				
**Morphological groups**							
*Leaf*							
	Substrate type	1	0.775	0.775	5.147	**0.013**	0.39
	Residuals	8	1.204	0.150			
	Total	9	1.979				
*Rhizome*							
	Substrate type	1	0.079	0.079	0.347	0.606	0.04
	Residuals	8	1.814	0.227			
	Total	9	1.893				

*Significant results are in bold.*

**TABLE 4:** Results of Simper analysis showing the taxa and species, and algal morphological groups most contributing to the multivariate pattern of the percentage cover of epiphytes on leaves of five-year old *Posidonia oceanica* seedlings grown on rock and sand substrate.

	**Species**	**Rock average abundance (%)**	**Sand average abundance (%)**	**Cumulative Contribution (%)**
**Taxa or species**				
*Leaf*				
	Encrusting	49.94	9.27	0.742
	*V. subulifera*	5.62	0.00	0.884
	*C. Dasyphylla*	0.35	2.87	0.947
	*O. caliculata*	0.00	0.99	0.969
	*D. mediterranea*	0.00	0.38	0.977
	*S. cirrosa*	0.32	0.05	0.985
	*C. mairei*	0.23	0.00	0.988
	Foraminifera	0.09	0.10	0.991
	*H. tenella*	0.08	0.00	0.993
	*O. geniculata*	0.06	0.00	0.994
	*D. fasciola*	0.06	0.00	0.996
	*Spirobis spp.*	0.01	0.06	0.997
	*Polysiphonia spp.*	0.00	0.05	0.998
	*H. secunda*	0.00	0.04	0.999
	*Ceramium sp.*	0.00	0.04	1
**Morphological groups**				
*Leaf*				
	Encrusting	49.94	9.27	0.774
	Filamentous	6.03	0.18	0.935
	Corticated	0.57	2.87	1

Multivariate analysis performed on the percentage cover of algal morphological groups confirmed that the structure of the leaf epiphyte community on rock was statistically different from that on sand (Table 3). Simper analysis showed that encrusting algae accounted for most of the differences observed between the two communities (76%; Table 4).

**FIGURE 4:**
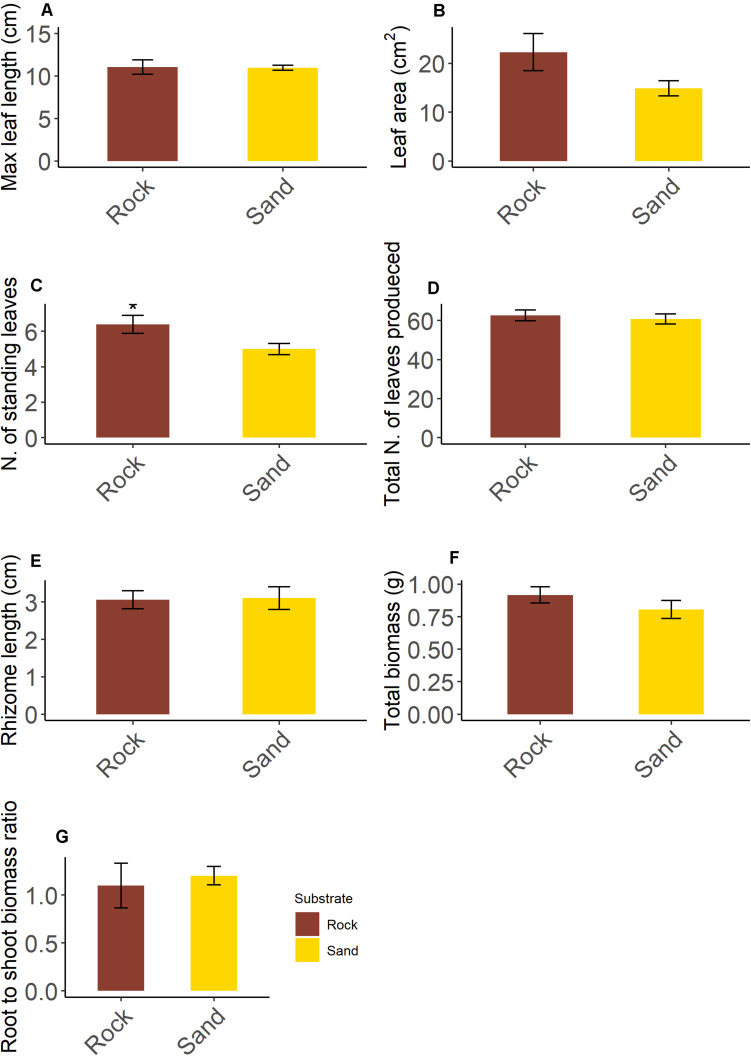
Morphological and growth variables of five-year old *Posidonia oceanica* seedlings grown on rock and sand substrate **(AG)**. Asterisk indicates a significant statistical difference at < 0.05. Data are means SE; *n* = 5.

Seedlings showed a rhizome with plagiotropic growth orientation bearing generally one shoot. However, up to three and two aborted shoots were observed on seedlings grown on rock and sand respectively. For most variables (Figure 4), no significant differences were detected among seedlings grown on rock and sand (leaf length, *t* = 0.234, df = 8, *p* = 0.814; leaf area, *t* = 1.801, df = 8, *p* = 0.109; total number of leaves produced during the life span, *t* = 0.476, df = 8, *p* = 0.647; rhizome length, *t* = 0.119, df = 8, *p* = 0.908; total plant biomass, *t* = 1.205, df = 8, *p* = 0.263; root to shoot biomass ratio, *t* = 0.412, df = 8, *p* = 0.691). However, seedlings on rock had more standing leaves than those on sand (*t* = 2.333, df = 8, *p* = 0.048): this difference was mainly due to a higher number of intermediate leaves. No correlation between epiphytic leaf cover and seedling growth variables was detected by RELATE procedure (Rock: Rho = 0.418, *p* = 0.843; Sand: Rho = 0.152 *p* = 0.686).

## Discussion

Our results support the hypothesis that seedlings grown on rock and sand differed in epiphyte cover and community structure, but only for leaves and not for rhizomes. Contrary to expectations, we did not observe differences in epiphyte abundance between the two sides of leaves. Also, no substantial differences in growth characteristics were found among seedlings grown on rock and sand, apart from the number of standing leaves. No significant correlation was detected among plant variables and epiphyte communities.

### Epiphyte Community of Leaves and Rhizomes of Seedlings

In total, we found a number of epiphytic organisms growing on *P. oceanica* seedlings within the range of that reported in previous studies (range: 1771; Supplementary Table 1 and references therein) on adult shoots collected in the same season (summer) and at similar depths (<5 m; Supplementary Table 1) to that of our study. Encrusting algae were the most abundant group present on the leaves of seedlings (contributing with more than 70% in cover on average), with *P. fragile* and *H. farinosum* as the main species belonging to this group. This is in accordance with previous studies on leaves of *P. oceanica* shoots (Supplementary Table 1) showing that encrusting algae was the most abundant group regardless of geographical area (e.g., Western and Eastern Mediterranean; Pardi et al., 2006; Ben Brahim et al., 2014b; Piazzi et al., 2016), plant age (e.g., seedling and adult plants; Celdran et al., 2012; Piazzi et al., 2016), and depth (e.g., shallow and deep meadows; Tsirika et al., 2007; Ben Brahim et al., 2014a; Piazzi et al., 2016). Encrusting algae have been also observed in one-year old *P. oceanica* seedlings (Celdran et al., 2012). It seems that the recruitment and growth of these algae on *P. oceanica* seedlings may be facilitated by the colonization of leaves by *Marinomonas posidonica*, a bacterium that exclusively grows on this species (Celdran et al., 2012).

We also found that epiphytes colonizing rhizomes of seedlings had an overall lower cover with respect to leaves, but no single taxon dominated the community as in leaves. Indeed, Clorophyta, Heterokontophyta, and Bryozoans were well represented on rhizomes while encrusting algae, Porifera, and Tunicata were absent. This finding is partially in accordance with that of previous studies on rhizomes of adult *P. oceanica* (Piazzi et al., 2016; Supplementary Table 1). The higher species diversity on rhizomes of adult plants (Piazzi et al., 2016) has been related to a more stable and sheltered environment offered by rhizomes to epiphytes that could allow the development of slow-growing but longer-living organisms.

In adult plants, epiphytes collected in summer at shallow depths (45 m) preferentially grow either on the external leaf side (Montefalcone et al., 2006) or the internal side (Peirano et al., 2011). These differences in epiphyte composition and abundance between the two leaf sides have been related to the shape and orientation of leaves in adult shoots (Van der Ben, 1971; Alcoverro et al., 2004; Peirano et al., 2011) and to the different light incidence and degree of exposure of each leaf side to hydrodynamics (Casola and Scardi, 1989; Tursi et al., 2001; Ben Brahim et al., 2014b; Piazzi et al., 2016). In our study, similarly to adult plants leaves of five-years-old seedlings showed the internal side concave and the external side convex. Yet, in contrast to adult plants we found a lack of preferential distribution of epiphytes between leaf sides. This contrasting result might be explained by the curvilinear shape and reduced length of leaves of seedlings with respect to adult leaves (i.e., tens of centimeters *vs.* 1 m, respectively; Boudouresque and Meinesz, 1982), which could lead to a similar degree of exposure of external and internal leaf sides to light intensity and hydrodynamics. Moreover, adult *P. oceanica* plants form densely packed shoots in which the internal leaf side can be less exposed to light and nutrients than the external one (Montefalcone et al., 2006; Piazzi et al., 2016). Thus, since in our study seedlings grew as a single plant bearing only one shoot, the external and internal leaf sides were probably similarly exposed to light and nutrients possibly explaining the lack of differences in epiphytes growth between the two leaf sides. Furthermore, in adult plants Briozoan, and *Electra posidoniae* especially, which account for a large portion of leaf epiphyte cover (Peirano et al., 2011; Lepoint et al., 2014a), are more abundant on the internal leaf side (Lepoint et al., 2014b). Surprisingly, however, in our study Bryozoan were absent on the leaves of seedlings, and only one species was found on rhizomes (*Amanthia lentigera*). This might further explain the similarity in epiphyte cover between leaf sides observed here in seedlings.

Bryozoan is one of the most represented epiphyte taxa found in adult plants, accounting for up to 15% of all epiphyte biomass (Piazzi et al., 2016). In particular, the bryozoan *E. posidoniae* grows exclusively on *P. oceanica*, and it is by far the most common species colonizing leaves of adult plants (up to 60% of all Bryozoan; Lepoint et al., 2014a,b). Thus, the absence of this species on leaves of seedlings found in our study is strikingly. However, the presence of Bryozoan on *P. oceanica* leaves is tightly associated with seasonal blooms of microphytoplancton (e.g., diatoms) in the water column, on which Bryozoans feed (Lepoint et al., 2014a,b). Both Bryozoan abundance and diversity increase in late winterearly spring, when food sources are abundant, and they strongly decline in summer due to food shortage (Lepoint et al., 2014a,b). In addition, Bryozoan follow a depth distribution gradient, with higher abundance and diversity on plants at deeper meadows (more than 10 m; Lepoint et al., 2014a,b) than the seedlings used in our study. Therefore, in this study the lack of Bryozoan on seedlings could be related to both the time of sampling (July) and habitat depth (less than 2 m).

### Effects of Substrate Type on the Epiphyte Community of Seedlings

The total leaf epiphyte cover we found on seedlings is in accordance with that of adult plants (6080%; Pardi et al., 2006). Yet, we found higher total epiphyte cover on seedlings grown on rock (60%) than on sand (13%) substrate, which disagrees with studies on adult plants (Giovannetti et al., 2008). Thus, our findings support the hypothesis that the nature of substrate or microhabitat conditions can drive the development of the seedling epiphyte community, as shown for the seagrasses *Thalassodendrum ciliatum* and *Posidonia sinuosa* (Cambridge and Kuo) which harbored a higher leaf epiphyte cover on plants growing on rock or closer to reef banks (Bandeira, 2002; Van Elven et al., 2004). Furthermore, in our study the difference observed in the leaf epiphyte community between seedlings that grew on rock and sand was mainly related to the higher presence of encrusting algae on rock. An experimental study on six-months old *P. oceanica* seedlings showed that the bacterium *M. posidonica*, which exclusively grows on *P. oceanica*, facilitated the recruitment and growth of encrusting algae (Celdran et al., 2012). Therefore, it is possible that the rock substrate not only provides for more propagules for algae recruitment, but it might also facilitate the development of specific bacteria capable to shape the epiphyte community.

In rhizomes, however, we did not detect any difference in epiphyte cover and community structure on seedlings between substrate types. This finding contrasts with studies which indicate that hard substrates enhance epiphyte richness on plants both for seagrass in general (Bandeira, 2002; Van Elven et al., 2004) and in particular for *P. oceanica* adult rhizomes (Eugene, 1979; Pessani et al., 1989). Rhizomes of seedlings were much smaller than those of adult plants and thus, the less space available for colonization might have prevented the development of different communities between substrates. More studies are needed to fully elucidate the mechanisms by which rocky substrates influence epiphyte growth on *P. oceanica* seedlings.

### Growth Characteristics of Seedlings

Our study shows that seedlings had produced approximately 60 leaves in five years. This corresponds to an average leaf production rate of about 12 leaves per year. This finding is in accordance with the leaf production rate previously observed in one-year old seedlings grown in natural environment (15 leaves per year; Balestri and Lardicci, 2008) and in aquarium (1214 leaves per year; Alagna et al., 2015). Interestingly, the number of standing leaves seems not to depend on the period of observation or the growing conditions (Balestri and Bertini, 2003; Balestri and Lardicci, 2008; Balestri et al., 2009; Alagna et al., 2013, 2015) as it remains fairly constant at least up to five years (in this study). The higher number of standing leaves of seedlings grown on rock suggests that, at least during the period of observation, the leaf turnover could differ due to variations in micro-habitat conditions. Yet, the number of standing leaves were lower than that observed in adult shoots at 5 m depth, ranging from 6 to 10 per shoot (Buia et al., 1992).

In this study, seedling rhizomes slowly grew in five years, reaching approximately 3 cm in length which corresponds to an average elongation rate of 0.6 cm per year. This is in contrast with previous studies on *P. oceanica* seedlings reporting a rhizome length of 3 cm after the first year and three years of growth (Balestri et al., 1998, 2009). In adult *P. oceanica* plants rhizomes can grow up to 6 cm per year (Marb and Duarte, 1998), which is an order of magnitude higher than what we found in this study. This is because adult plants can benefit from clonal integration which provides them the necessary resources for sustaining such a high growth rate (Ruocco et al., 2021).

Contrary to expectations, seedlings grown on rock and sand showed similar growth characteristics, except for the number of standing leaves. This finding contrasts with previous studies showing both higher leaf and rhizome length in one-year old *P. oceanica* seedlings growing on rock than on sand (Alagna et al., 2015, 2020). However, these studies were conducted in aquaria and on very short temporal scales. It is plausible that during their first year, seedlings on sand invest more biomass in roots at the expense of shoots to avoid uprooting (Infantes et al., 2011; Alagna et al., 2015). Instead, in our study the root to shoot biomass ratio of seedlings had approximately a value of one, indicating that they had equally invested on shoots and roots regardless of substrate type. In addition, in natural habitats other factors (e.g., light and nutrients) might have interacted with substrate in influencing seedling development.

## Conclusion

Seagrass epiphytes have been widely studied but virtually nothing is known about the factors driving the recruitment and structure of epiphyte communities on seedlings. The present study provides valuable insights on the epiphytic community on five-year old of seedlings of *P. oceanica* grown on different types of micro-habitat (rock fissures and sand patches). It also provides new data on the growth potential of *P. oceanica* seedlings. Specifically, we showed that despite their small size, *P. oceanica* seedlings hosted an epiphyte community relatively well-structured and largely reflecting that of adult plants, although with some notable differences, i.e., the absence of Bryozoans, Porifera, and Tunicata, and lack of a preferential distribution on the external leaf side. Interestingly, our study suggests that micro-habitat (i.e., substrate type) can influence the structure and cover of leaf epiphyte communities on seedlings, but it does not substantially affect seedling growth and biomass allocation. Furthermore, the lack of correlation between plant growth variables and epiphyte communities across substrates emerged from this study suggests that plants characteristics are not strongly involved in shaping the epiphyte communities. Overall, these findings increase our understanding of the biological interactions occurring during the most critical life history stage of this seagrass. Considering the importance that seagrass seedlings may play in colonization, recovery after disturbances, as well as in restoration efforts, further studies are needed to elucidate possible positive or negative effects of epiphyte cover on seedling growth; for instance, through the transfer of or competition for nutrients or the increase in drag forces on seedlings leading to higher dislodgement.

## Data Availability Statement

The raw data supporting the conclusions of this article will be made available by the authors, without undue reservation.

## Author Contributions

DDB performed seedling morphological characterization and statistical analyses, interpreted results, and wrote the manuscript. EB designed the study, supervised the experiment, performed seedling morphological characterization and statistical analyses, interpreted the results, and wrote the manuscript. GP performed seedling epiphyte characterization and contributed to wrote the manuscript. VM contributed to statistical analyses and to wrote the manuscript. CL designed the study, supervised the experiment, and revised the manuscript. All authors read and approved the final version of the manuscript.

## Conflict of Interest

The authors declare that the research was conducted in the absence of any commercial or financial relationships that could be construed as a potential conflict of interest.
