# Supplementary material for: Substrate Type Influences the Structure of Epiphyte Communities and the Growth of Posidonia oceanica Seedlings
Source: Front Plant Sci. 2021 May 7;12:660658. doi: 10.3389/fpls.2021.660658 (PMC8139341; doi:10.3389/fpls.2021.660658)
Supplement: Supplementary file 1 [file Data_Sheet_1.docx]

Supplementary Material

**
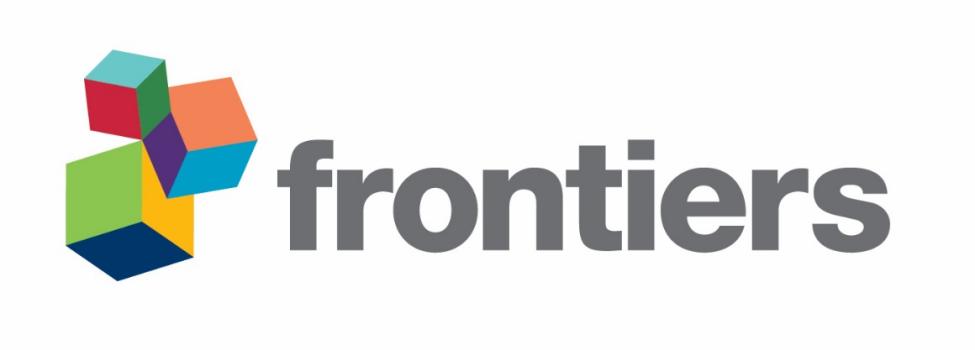
**


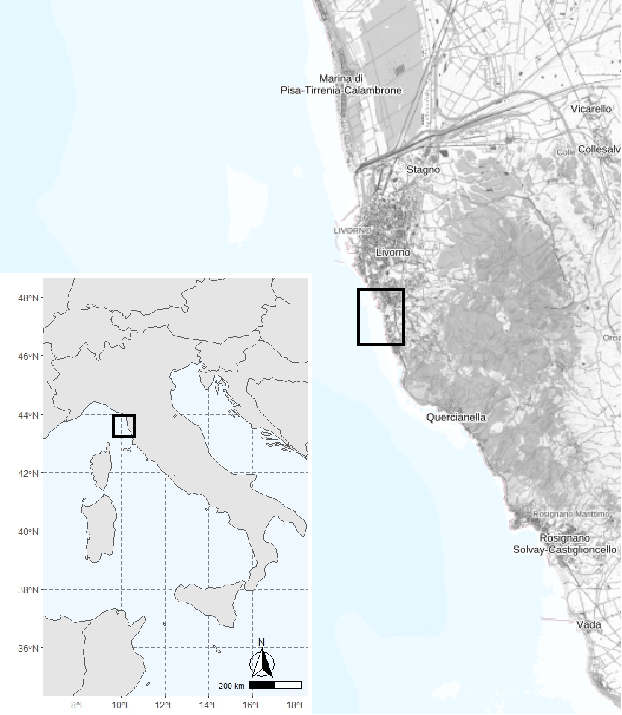


Figure 1 - Area of *Posidonia oceanica* seeds collection and seedlings transplantation. Seedlings were transplanted at 1.5 meters depth, on rocks and patches of medium-fine calcareous sand.

Table S1 - Summary of the epiphyte species found on Posidonia oceanica leaves or rhizomes at shallow depths (<5 m). () indicates that the species was not recorded in the Western Mediterranean according to Piazzi et al., 2016. In bold, species also found on seedlings in our study are highlighted.

| Species | Leaf | Rhizome | Reference |
| --- | --- | --- | --- |
| **Heterokontophyta** |  |  |  |
| *Apoglossum ruscifolium* (Turner) J.Agardh | yes | no | Ben Brahim et al., 2014b; Mabrouk et al., 2013 |
| *Asperococcus bullosus* J.V. Lamouroux | yes | no | Balata et al., 2009 |
| *Asperococcus sp.* | yes | no | Ben Brahim et al., 2014b; Mabrouk et al., 2013 |
| *Castagna sp* | yes | no | Mabrouk et al., 2013 |
| *Cladosiphon cylindricus* (Sauvageau) Kylin | yes | no | Ben Brahim et al., 2014; Ben Brahim et al., 2014b |
| *Cladosiphon irregularis* (Sauvageau) Kylin | yes | no | Balata et al., 2009 |
| *Cladosiphon mediterraneus* Kutzing | yes | no | Tsirika et al., 2007 |
| *Cutleria multifida* (J.E. Smith) Greville | yes | yes | Ben Brahim et al., 2014b; Mabrouk et al., 2013, 2013b |
| *Cystoseira crinita* (Duby | no | yes | Mabrouk et al., 2013b |
| *Cystoseira foeniculacea* f. schiffneri  (Hamel) Gomez Garreta, Barcel o,  Ribera & Rull Lluch | no | yes | Mabrouk et al., 2013b |
| *Dictyopteris polypodioides* (A.P.De Candolle) J.V.Lamouroux) | (yes) | yes | Ben Brahim et al., 2014; Mabrouk et al., 2013b |
| *Dictyota dichotoma* (Hudson) J.V Lamouroux | yes | yes | Ben Brahim et al., 2014b; Mabrouk et al., 2013b |
| *Dictyota linearis* (C. Agardh) Greville | yes | yes | Ben Brahim et al., 2014b; Mabrouk et al., 2013, 2013b; Tsirika et al., 2007 |
| *Ectocarpus siliculosus* (Dillwyn) Lyngbye | yes | no | Ben Brahim et al., 2014b; Mabrouk et al., 2013 |
| *Giraudya sphacelarioides* (Derbes et Solier) | yes | no | Ben Brahim et al., 2014; Mabrouk et al., 2013; Tsirika et al., 2007 |
| *Myrionema orbiculare* (Agardh) | yes | no | Ben Brahim et al., 2014; Giovannetti et al., 2008; Mabrouk et al., 2013 |
| *Padina pavonica* (Linnaeus) Thivy | no | yes | Mabrouk et al., 2013b |
| ***Sphacelaria cirrosa*** (Roth) C.Agardh 1824 | yes | yes | Ben Brahim et al., 2014; Tsirika et al., 2007; Mabrouk et al., 2013b |
| *Sphacelaria rigidula* Kutzing | yes | no | Tsirika et al., 2007 |
| *Sphacelaria tribuloides* Meneghini | yes | no | Ben Brahim et al., 2014b; Mabrouk et al., 2013 |
| **Rhodophyta** |  |  |  |
| *Aglaothamnion scopulorum* (C. Agardh) Feldmann-Mazoyer | yes | no | Ben Brahim et al., 2014b; Mabrouk et al., 2013 |
| *Aglaothamnion tenuissimum* | yes | no | B.Martínez-Crego et al., 2010 |
| *Aglaothamnion tripinnatum* (C. Agardh) Feldmann-Mazoyer | yes | yes | Ben Brahim et al., 2014b; Mabrouk et al., 2013 |
| *Amphiroa rigida* (Lamouroux) | (yes) | yes | Ben Brahim et al., 2014b; Mabrouk et al., 2013, 2013b |
| *Antithamnion cruciatum* (C. Agardh) Nägeli | (yes) | no | B.Martínez-Crego et al., 2010; Ben Brahim et al., 2010, 2014b; Mabrouk et al., 2013 |
| ***Ceramium*** *codii* (Richards) Mazoyer | yes | yes | Ben Brahim et al., 2010, 2014b; Mabrouk et al., 2013; Tsirika et al., 2007 |
| ***Ceramium*** *diaphanum* (Lighfoot) Roth | yes | no | Tsirika et al., 2007 |
| ***Ceramium*** *flaccidum* (Harvey ex Ku¨ tzing) Ardissone | yes | no | B.Martínez-Crego et al., 2010; Tsirika et al., 2007 |
| ***Ceramium*** *gracillimum* (Kützing) Zanardini | yes | no | Ben Brahim et al., 2014b |
| ***Ceramium*** *tenuissimum* (Roth) Aresch | yes | no | Ben Brahim et al., 2010, 2014b; Mabrouk et al., 2013 |
| *Chondria capillaris* | yes | no | B. Martínez-Crego et al., 2010 |
| ***Chondria mairei*** Feldmann-Mazoyer | yes | no | Tsiriki et al., 2007 |
| *Chroodactylon ornatum* (C. Agardh) Basson | yes | no | Tsirika et al., 2007 |
| *Chylocladia verticillata* | yes | no | B. Martínez-Crego et al., 2010 |
| *Crouania attenuata* | yes | no | B. Martínez-Crego et al., 2010 |
| *Cryptonemia lomation* (Bertoloni) J.Agardh 1851 | (yes) | + | Ben Brahim et al., 2014, Mabrouk et al., 2013b |
| *Dasya corimbifera* | yes | no | B. Martínez-Crego et al., 2010 |
| *Dasya sp.* | yes | yes | Ben Brahim et al., 2010, 2014b |
| *Dasya rigidula* (Kutzing) Ardissone | yes | no | Tsirika et al., 2007 |
| *Erythritrichia carnea* (Dillwyn) J. Agardh | yes | no | Tsirika et al., 2007 |
| *Falkenbergia rufolanosa* (Harvey) F. Schmitz | yes | yes | Ben Brahim et al., 2014b |
| *Feldmania globifera* (Kuetz.) Hamel | yes | no | Ben Brahim et al., 2014b; Mabrouk et al., 2013 |
| *Fosliella sp.* | yes | no | Ben Brahim et al., 2014; Mabrouk et al., 2013 |
| *Griffithsia opuntioides* J. Agardh | yes | yes | Ben Brahim et al., 2014b |
| *Gymnothamnion elegans* (Schousboe ex C. Agardh) J. Agardh | yes | no | Tsiriki et al., 2007 |
| ***Herposiphonia secunda*** (C. Agardh) Ambronn | yes | no | Tsiriki et al., 2007 |
| ***Herposiphonia tenella*** (C. Agardh) Ambronn | yes | no | Tsirika et al., 2007 |
| *Heterosiphonia crispella* (C. Agardh) Wynne | yes | yes | Ben Brahim et al., 2014b; Mabrouk et al., 2013; Tsirika et al., 2007 |
| ***Hydrolithon farinosum*** (Lamouroux) Penrose et Chamberlain | yes | no | Ben Brahim et al., 2014b; Mabrouk et al., 2013, 2013b; Tsirika et al., 2007 |
| *Hypnea musciformis* (Wulfen) J. V. Lamouroux | no | yes | Mabrouk et al., 2013b |
| *Jania rubens* (Linnaeus) J. V. Lamouroux | no | yes | Mabrouk et al., 2013b |
| *Laurencia minuta* Vandermeulen, Garbary et Guiry | yes | no | Tsirika et al., 2007 |
| *Laurencia obtusa* (Hudson) J.V. Lamouroux | yes | yes | Ben Brahim et al., 2014b |
| *Lejolisia mediterranea* Bornet | yes | no | Tsirika et al., 2007 |
| *Lithophyllum incrustans* R.A.Philippi | yes | yes | Ben Brahim et al., 2014; Mabrouk et al., 2013b |
| *Lithophyllum racemus* (Lamarck) Foslie 1901 | (yes) | yes | Ben Brahim et al., 2014; Mabrouk et al., 2013b |
| ***Lophosiphonia obscura*** (C. Agardh) Falkenberg | yes | no | Tsirika et al., 2007 |
| *Neomonospora sp.* | (yes) | no | Ben Brahim et al., 2014b |
| *Peyssonnelia squamaria* (S.G.Gmelin) Decaisne 1842 | (yes) | yes | Ben Brahim et al., 2014; Mabrouk et al., 2013b |
| *Phymatolithon calcareum* (Pallas) W.H.Adey & D.L.McKibbin 1970 | (yes) | yes | Ben Brahim et al., 2014; Mabrouk et al., 2013b |
| *Phymatolithon lenormandii* (Areschoug) W.H.Adey 1966 | (yes) | yes | Ben Brahim et al., 2014; Mabrouk et al., 2013b |
| *Platythamnion sp.* | yes | no | Ben Brahim et al., 2014b; Mabrouk et al., 2013 |
| *Pleonosporium borreri* (J.E. Smith) Nägeli | (yes) | yes | Ben Brahim et al., 2010, 2014b |
| ***Pneophyllum fragile*** (Kützing) | (yes) | yes | Ben Brahim et al., 2014b; Mabrouk et al., 2013, 2013b; Tsirika et al., 2007 |
| *Polysiphonia breviarticulata* (C. Agardh) Zanardini | yes | no | Tsirika et al., 2007 |
| *Polysiphonia elongata* (Hudson) Sprengel | (yes) | yes | Ben Brahim et al., 2010, 2014b; Mabrouk et al., 2013, 2013b |
| *Polysiphonia flocculosa* ( Agardh) Endliche | yes | no | Ben Brahim et al., 2014b; Mabrouk et al., 2013 |
| *Polysiphonia scopulorum* Harvey | yes | no | Tsirika et al., 2007 |
| ***Polysiphonia sp.*** | yes | yes | B. Martínez-Crego et al., 2010 |
| *Rhodymenia sp.* | no | yes | Mabrouk et al., 2013b |
| *Spermothamnion flabellatum* (Bornet) | yes | no | Ben Brahim et al., 2014b; Mabrouk et al., 2013 |
| *Spermothamnion repens* (Dillwyn) Rosenvinge | yes | no | Tsirika et al., 2007 |
| *Stylonema alsidii* (Zanardini) K.M. Drew | yes | no | Tsirika et al., 2007 |
| **Chlorophyta** |  |  | Ben Brahim et al., 2014b |
| *Anadyomene stellata* | no | yes | Mabrouk et al., 2013b |
| *Caulerpa prolifera* (Forsskal) J. V. Lamouroux | no | yes | Mabrouk et al., 2013b |
| *Cladophora dalmatica* | yes | no | B. Martínez-Crego et al., 2010 |
| *Cladophora prolifera* (Roth) Kützing | no | yes | Balata et al., 2009 |
| *Cladophora sp.* | yes | yes | Ben Brahim et al., 2014b; Tsirika et al., 2007 |
| *Dasycladus vermicularis* (Scopoli) Krasser | (yes) | no | Ben Brahim et al., 2010, 2014b; Mabrouk et al., 2013 |
| *Flabellia patiolata* (Turra) Nizamuddin | no | yes | Mabrouk et al., 2013 |
| *Halimeda tuna* (J. Ellis & Solander) J. V. Lamouroux | no | yes | Mabrouk et al., 2013 |
| *Phaeophila dendroides* (P. et H. Crouan) Batters | yes | no | Tsirika et al., 2007 |
| *Siphonocladus pusillus* (C. Agardh ex Kützing) Hauck | (yes) | no | Ben Brahim et al., 2014b; Mabrouk et al., 2013 |
| **Bryozoa** |  |  | Ben Brahim et al., 2014b |
| *Aetea sica* | yes | no | B. Martínez-Crego et al., 2010 |
| *Aetea truncata* Landsborough | yes | yes | Ben Brahim et al., 2010, 2014b; Mabrouk et al., 2013b |
| *Alcyonidium gelatinosum* (Linnaeus) | no | yes | Mabrouk et al., 2013b |
| *Alcyonidium sp.* | (yes) | no | Ben Brahim et al., 2014b |
| ***Amathia lentigera*** Linnaeus | yes | yes | Ben Brahim et al., 2014b; Mabrouk et al., 2013b |
| *Beania hirtissima* (Heller) | yes | yes | Ben Brahim et al., 2014b; Mabrouk et al., 2013b |
| *Beania magellanica* Bush | yes | yes | Ben Brahim et al., 2014 |
| *Bowerbankia imbricata* Adams | (yes) | no | Ben Brahim et al., 2010, 2014b |
| *Calpensia nobilis* (Esper) | no | yes | Mabrouk et al., 2013b |
| *Cellaria salicornoides* Lamouroux | (yes) | yes | Ben Brahim et al., 2014 |
| *Cellaria sinuosa* (Hassall) | no | yes | Mabrouk et al., 2013b |
| *Chelidonia cordieri* (Audouin) | yes | no | Ben Brahim et al., 2010, 2014b |
| *Chorizopora brogniartii* (Audouin) | yes | yes | Ben Brahim et al., 2014b; Mabrouk et al., 2013 |
| *Cribrilina radiata* (Smitt) | yes | yes | Ben Brahim et al., 2010, 2014b |
| *Electra pilosa* | yes | no | B. Martínez-Crego et al., 2010 |
| *Electra posidoniae* (Gautier) | yes | no | Ben Brahim et al., 2010, 2014b; Giovannetti et al., 2008; Mabrouk et al., 2013 |
| *Fenestrulina malusii* (Audouin) | yes | yes | Ben Brahim et al., 2014b; Mabrouk et al., 2013 |
| *Frondipora verrucosa* (Lamouroux) | no | yes | Mabrouk et al., 2013b |
| *Lichenopora radiata* (Audouin) | yes | yes | Ben Brahim et al., 2010, 2014b; Mabrouk et al., 2013, 2013b |
| *Membranipora membranacea* (Linnaeus) | no | yes | Mabrouk et al., 2013b |
| *Micropora complanata* (Gautier) | yes | no | Ben Brahim et al., 2010, 2014b; Mabrouk et al., 2013 |
| *Microporella sp.* | yes | yes | Ben Brahim et al., 2014b; Mabrouk et al., 2013 |
| *Schizobrachiella sanguinea* Norman | no | yes | Balata et al., 2009; Mabrouk et al., 2013b |
| *Scrupocellaria sp.* | (yes) | yes | Ben Brahim et al., 2010, 2014b |
| *Tubulipora flabellaris* Fabricius | yes | yes | Mabrouk et al., 2013b |
| *Tubulipora plumosa* Thompson in Harmer | no | yes | Mabrouk et al., 2013b |
| *Tubulipora sp.* | yes | yes | Ben Brahim et al., 2014b; Mabrouk et al., 2013 |
| **Hydrozoa** |  |  | Ben Brahim et al., 2014b |
| *Aglaophenia sp.* | yes | yes | Ben Brahim et al., 2010, 2014b; Mabrouk et al., 2013 |
| *Campanularia hincksi* Alder | (yes) | yes | Ben Brahim et al., 2010, 2014b |
| *Dynamena cavolinii* Neppi | yes | no | Balata et al., 2009; Ben Brahim et al., 2010, 2014b; Mabrouk et al., 2013 |
| *Gonothyraea gracilis* M. Sars | yes | no | Ben Brahim et al., 2014b |
| *Halocordyle disticha* Goldfuss | yes | no | Ben Brahim et al., 2010, 2014b |
| *Monotheca obliqua* | yes | no | Giovannetti et al., 2008 |
| *Monotheca sp.* | yes | no | Ben Brahim et al., 2010, 2014b |
| ***Obelia geniculata*** Linnaeus | yes | no | Ben Brahim et al., 2010, 2014b; Mabrouk et al., 2013 |
| ***Orthopyxis caliculata*** Hincks | yes | no | Ben Brahim et al., 2010, 2014b |
| *Plumularia obliqua* Thompson | yes | no | B. Martínez-Crego et al., 2010 |
| *Plumularia posidoniae* | yes | no | B. Martínez-Crego et al., 2010 |
| *Plumularia setacea* Linnaeus | (yes) | no | Ben Brahim et al., 2010, 2014b |
| *Sertularia perpusilla* Stechow | yes | yes | B. Martínez-Crego et al., 2010; Ben Brahim et al., 2014b; Giovannetti et al., 2008; Mabrouk et al., 2013 |
| **Annelida** |  |  |  |
| *Brania clavata* Claparede | yes | no | Ben Brahim et al., 2014b |
| *Janua pagenstecheri* (Quatrefageau) | yes | yes | Ben Brahim et al., 2014b; Mabrouk et al., 2013 |
| *Serpula vermicularis* (Linnaeus) | no | yes | Mabrouk et al., 2013b |
| *Serpulidae* | (yes) | no | Giovannetti et al., 2008 |
| *Spirobranchus triqueter* (Linnaeus) | no | yes | Mabrouk et al., 2013b |
| *Spirorbis spirorbis* Linnaeus | yes | no | Ben Brahim et al., 2010, 2014b; Mabrouk et al., 2013 |
| ***Spirorbis sp.*** | no | yes | Mabrouk et al., 2013b |
| *Vermiliopsis infundibulum* (Philippi) | no | yes | Mabrouk et al., 2013b |
| **Porifera** |  |  |  |
| *Clathrina coriacea* (Montagu) | no | yes | Mabrouk et al., 2013b |
| *Dysidea avara* (Schmidt) | no | yes | Mabrouk et al., 2013b |
| *Halichondria sp.* | yes | no | Ben Brahim et al., 2014b |
| *Haliclona simulans* (Johnston) | no | yes | Mabrouk et al., 2013b |
| *Hemimycale columella* (Bowerbank) | no | yes | Mabrouk et al., 2013b |
| *Ircinia dendroides* (Schmidt) | no | yes | Mabrouk et al., 2013b |
| *Ircinia fasciculata* Esper | yes | no | Ben Brahim et al., 2014b |
| *Ircinia muscarum* Schmidt | yes | no | Ben Brahim et al., 2014b |
| *Ircinia oros* (Schmidt) | no | yes | Mabrouk et al., 2013b |
| *Ircinia variabilis* (Schmidt, 1862) | no | yes | Mabrouk et al., 2013b |
| *Scalarispongia sp.* | no | yes | Mabrouk et al., 2013b |
| **Tunicata** |  |  |  |
| *Aplidium conicum* (Olivi) | no | yes |  |
| *Botryllus schlosseri* Pallas | yes | no | Ben Brahim et al., 2014b |
| *Clavelina lepadiformis* (light-bulb sea squirt) | yes | no | Ben Brahim et al., 2014b; Mabrouk et al., 2013b |
| *Didemnum maculosum* (Milne-Edwards) | no | yes | Mabrouk et al., 2013b |
| *Diplosoma listerianum* (Milne-Edwards) | no | yes | Mabrouk et al., 2013b |
| *Phallusia nigra* (Savigny) | no | yes | Mabrouk et al., 2013b |
| *Polysyncraton lacazei* (Giard) | no | yes | Mabrouk et al., 2013b |
| *Trididemnum cereum* (Giard) | no | yes | Mabrouk et al., 2013b |
